# A Method to Generate and Rescue Recombinant Adenovirus Devoid of Replication-Competent Particles in Animal-Origin-Free Culture Medium

**DOI:** 10.3390/v15112152

**Published:** 2023-10-25

**Authors:** Seyyed Mehdy Elahi, Jennifer Jiang, Nazila Nazemi-Moghaddam, Rénald Gilbert

**Affiliations:** 1 Department of Production Platforms & Analytics, National Research Council Canada, Building Montreal, 6100 Avenue Royalmount, Montreal, QC H4P 2R2, Canada; jennifer.jiang2@mail.mcgill.ca (J.J.); nazila.nazemi-moghaddam@cnrc-nrc.gc.ca (N.N.-M.); renald.gilbert@cnrc-nrc.gc.ca (R.G.); 2 Department of Bioengineering, McGill University, Montreal, QC H3A 0E9, Canada

**Keywords:** adenovirus vector, replication-competent adenovirus, limiting dilution, animal-origin-free culture media

## Abstract

Adenoviruses are promising vectors for vaccine production and gene therapy. Despite all the efforts in removing animal-derived components such as fetal bovine serum (FBS) during the production of adenovirus vector (AdV), FBS is still frequently employed in the early stages of production. Conventionally, first-generation AdVs (E1 deleted) are generated in different variants of adherent HEK293 cells, and plaque purification (if needed) is performed in adherent cell lines in the presence of FBS. In this study, we generated an AdV stock in SF-BMAdR (A549 cells adapted to suspension culture in serum-free medium). We also developed a limiting dilution method using the same cell line to replace the plaque purification assay. By combining these two technologies, we were able to completely remove the need for FBS from the process of generating and producing AdVs. In addition, we demonstrated that the purified AdV stock is free of any replication-competent adenovirus (RCA). Furthermore, we demonstrated that our limiting dilution method could effectively rescue an AdV from a stock that is highly contaminated with RCA.

## 1. Introduction

The replicative defective adenovirus vector (RD-AdV) with a deletion in its E1 region is one of the most useful adenovirus vectors for gene therapy and vaccination (as reviewed: [1,2,3]). Furthermore, since the beginning of the COVID-19 pandemic, AdVs have been the most common type of viral vector used for the production of COVID-19 vaccines [4]. To increase the safety of vaccines and gene therapies, the virus is typically rendered replication-incompetent by deleting its E1 gene. In a typical E1-deleted AdV, the gene of interest (GOI) is carried by the vector in place of the essential E1 region (E1A and E1B genes). Amplification of this type of AdV must take place in complementing cell lines, such as HEK293A cells (an adherent version of HEK293), that provide the E1 functions in *trans.* HEK293A cells were established by the stable transfection of human embryonic kidney cells with a segment of the adenovirus genome corresponding to the left end of the vector [5,6]. AdVs that possess flanking homology sequences with the adenovirus fragment are integrated into HEK293A cells, and this allows the acquisition of the E1 region through homologous recombination. This homologous recombination results in the emergence of replication-competent adenovirus (RCA) and the loss of GOI [7,8,9]. However, the presence of RCA in biological products, such as those used for gene therapy and vaccine development, is not desirable because it increases the product toxicity and reduces its potency. The presence of RCA is thus highly regulated. The maximum limit of RCA in biological products used for human application is 1 in 3 × 10^10^ viral particles [10]. Numerous efforts have been made to reduce or eliminate RCA. One of the approaches is to generate complementing cell lines that prevent the formation of RCA during vector growth. A few complementing cell lines have been generated with reduced homology between the integrated adenovirus sequence and the vector [11,12,13,14,15,16,17,18]. Three of these cell lines were also adapted to grow in suspension culture and in serum-free medium for easier scale-up during viral vector production and for regulatory compliance [11,12,19]. Previously, we have established such a complementing cell line, referred to as SF-BMAdR cells, by stable transfection of the minimal E1A and E1B genes needed for complementation into human lung carcinoma (A549), to prevent RCA formation by homologous recombination during recombinant AdV production. Despite the progress made in establishing new complementing cell lines, many scientists still use different variants of HEK293 (adherent or suspension) cell lines for the production of their recombinant adenoviruses since the cells are widely available with well-established experimental protocols.

Another issue in AdV production is the use of fetal bovine serum (FBS) in cell culture. The presence of FBS in the culture media is challenging and undesirable for clinical applications due to (I) its complex and variable composition, (II) the risk of contamination with animal viruses, and (III) ethical issues concerning animal cruelty [20]. The generation of AdV can be performed through homologous recombination in mammalian cells or by in vitro molecular cloning followed by transfection of the digested DNA into mammalian cells [1]. In both methods, FBS is normally added to the cell culture. Also, FBS is added during the plaque purification assay for isolating recombinant adenovirus from the parental virus (if recombination is performed in mammalian cell line) or for cloning the AdV.

To attempt to solve the current challenges surrounding the safety and purity requirements of AdV for clinical applications, in this study, we have developed a method to generate, isolate, and amplify clones of recombinant adenovirus devoid of RCA in the absence of FBS. In addition, we have demonstrated that the limiting dilution method developed in this study can be used to purify a recombinant adenovirus from a viral stock highly contaminated with RCA.

## 2. Materials and Methods

### 2.1. Cells Lines, Plasmids, and Viruses

Three stable cell lines were used in the current study: anchorage-dependent HEK293A cells [5] (HEK293A is another name for HEK293. The letter “A” in HEK293A cells refers to the adherent origin of cell line and differentiates it from HEK293S or HEK293SF that are adapted to suspension culture, in the presence or in the absence of FBS), A549 cells (a human lung carcinoma) (obtained from ATCC, CCL-185), and SF-BMAdR cells (produced in-house [12]). The HEK293A and A549 cell lines were grown in Dulbecco’s modified Eagle’s medium (Gibco, Life Technologies, Ottawa, ON, Canada), supplemented with 5% of fetal bovine serum (Hyclone, Logan, TU, USA) and 200 mM of glutamine (Gibco, Life Technologies, Ottawa, ON, Canada). The SF-BMAdR were grown in PRO293s CDM (PRO293s, Lonza, Walkersville, MD, USA) or Hycell TransFX H medium (Hycone, Logan, UT, USA) and were supplemented with 6 mM of glutamine, respectively. Adherent cells were grown in treated 96-well plates or 10 and 15 cm plates (Sarstedt, Numbrecht, North Rhine-Westphalia, Germany). Depending on the volume of cell culture, the suspension cultures were performed in 96-, 24-, and 6-well plates (Sarstedt, Numbrecht, North Rhine-Westphalia, Germany) or in 125 mL to 2 L shake flasks (Corning, Oneonta, NY, USA). Suspension culture agitation (for volume of ≥3 mL) was conducted at 100–120 rpm using orbital shakers (Bellco, or GL-300 Analytics).

The construction of pAdEasy-1-NS3-5a by the AdEasy plasmid system (Agilent Technologies, Santa Clara, CA, USA) which expresses hepatitis C virus NS3 to NS5a antigens was described previously [21]. This plasmid was used for the generation and large-scale production of Ad5-CMV5-NS3-5a in SF-BMAdR cell line without FBS. AdErB2, a first-generation AdV expressing the ErB2 gene [22], was used as a model for E1-deleted AdV. AdPTG3602 [23], a wild-type adenovirus without any deletion in the genome, was used as a surrogate for RCA.

### 2.2. Developing a Limiting Dilution Method to Replace Plaque Purification

**Figure 1:**
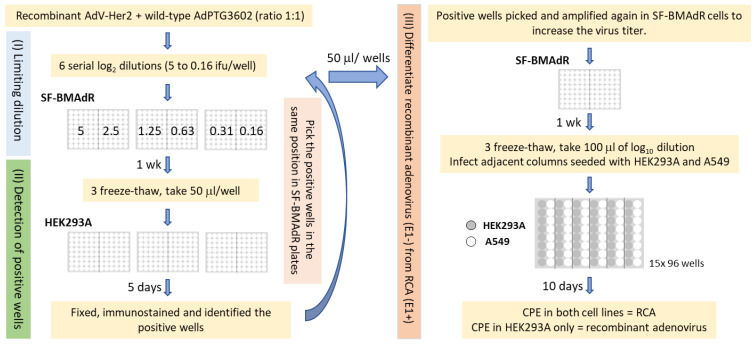
Workflow for rescuing recombinant adenoviruses from stock highly contaminated with RCA via limiting dilution and characterization of AdV clones. This protocol consists of three steps. (**I**) Limiting dilution step in which 48 wells were infected with 6 serial log_2_ dilutions (5 to 0.16 ifu) per well of the AdV/RCA mixed stock. (**II**) Detection step in which positive wells were identified by immunostaining using HEK293A cells. (**III**) Differentiation step in which, after one additional round of clone amplification in SF-BMAdR cells, each clone infects HEK293A and A549 cells in parallel to differentiate whether they are rAdV or RCA.

As the acceptable limit of RCA in biological material, in general, is extremely low, it is not possible to show the clonality and effectiveness of our limiting dilution method for isolating the AdV from RCA without artificially increasing the initial amounts of RCA in the test sample. Thus, an artificial AdV viral stock containing high amounts of wild-type adenovirus was generated to demonstrate the efficacy of our limiting dilution. The AdV:Her2 (as the surrogate for first-generation AdV), with wild-type adenovirus (as the surrogate for RCA) had a 1:1 ratio (Figure 1). Three 96-well plates were seeded with 30,000 SF-BMAdR cells in 50 μL/well. A total of 6 log_2_ dilutions of mixed viruses starting with 5 infectious units (ifu) to 0.16 ifu/50 μL was prepared. Immediately, 48 wells were infected with 50 μL of each log_2_ dilution of the mixed viruses. After 1 week of incubation, the plates underwent three rounds of freeze–thaw cycles. In the next step, the HEK293A cells were used to differentiate the infected wells from noninfected wells. Three plates of 96-well plates were seeded with 15,000 HEK293A cells/well and, the day after, plates were infected with 50 μL of the supernatant of infected SF-BMAdR cells. Each well on the infected plate of HEK293A corresponds to the same position/clone on the infected SF-BMAdR plates. Five days post-infection (p.i.), cells were fixed with methanol, and the presence of adenovirus fiber in infected cells was confirmed by immunostaining via a protocol described previously [24]. Briefly, 100% methanol was used as a fixing agent. PBS + 2% FBS was used as a washing and blocking reagent, as well as a diluent for the monoclonal antibodies used during immunostaining, and conjugated. The plates were washed three times between each step of the immunostaining. The fixed cells were blocked for 60 min, and incubated for 2 h with an in-house monoclonal antibody against adenovirus fiber (2G11-2) at a concentration of 1 µg/mL. Next, the samples were washed and incubated for 1 h at room temperature with a secondary antibody, which is a fluorescein isothiocyanate (FITC) conjugated anti-mouse antibody (Fluorescein-AffiniPure F(ab’)2 Fragment Goat Anti-Mouse IgG, Fc gamma Fragment Specific, (Jackson ImmunoResearch #115-096-071, West Grove, PA, USA)) used at a dilution of 1/600.

To differentiate recombinant adenovirus from RCA, the positive colonies from infected SF-BMAdR 96-well plates were picked and amplified once in SF-BMAdR cells via the same protocol as described above to increase virus titer. In the next step, 96-well plates were seeded with 15,000 cells/well of HEK293A and A549 cells in alternating columns. The day after, 50 μL of log_10_ dilution (from 10^−1^ to 10^−8^) of each amplified AdV clone was added to eight wells of one column for each cell line, with each dilution added to one well per cell line. The presence of a cytopathic effect (CPE) was observed and scored at 10 days p.i. in both cell lines using a bright-field microscope.

### 2.3. Generation of rAdV without FBS

A recombinant adenovirus encoding the HCV-NS3 to NS5a antigens (Ad5-CMV5-NS3-5a) was made previously by homologous recombination in bacteria using the AdEasy plasmid system (Agilent Technologies, Santa Clara, CA, USA). The virus was made by transfecting the HEK293A with a PacI-digested shuttle vector [21]. In this study, we repeated the experiment by replacing the HEK293A with SF-BMAdR cells. A total of 3 mL of SF-BMAdR cells in Pro293s medium was seeded in 6-well plates at a concentration of 10^6^ cells/mL and transfected with 3 or 5 μg of the PacI-digested shuttle vector using Polyethylenimine (PEIpro, Polyplus, Illkirch, France) with the ratio of PEIpro to DNA being 1.5 to 1. At 7 days post-transfection, the transfected cells were frozen and thawed three times. Supernatant of infected cells in both transfections was subject to two rounds of limiting dilution as described above, with only minor modifications. In the first round of limiting dilution, the starting dilution for the first log2 dilution was 2 × 10^−1^ and 32 wells were infected per dilution; in the second round of limiting dilution, the first dilution was 2 × 10^−2^ and 24 wells were infected for each dilution. After the first round of limiting dilution, one clone from each transfection was selected for the second round of limiting dilution. 

### 2.4. Scale-Up and RCA Detection

After the second round of limiting dilution of Ad5-CMV5-NS3-5a, 12 subclones were selected to be amplified in SF-BMAdR cells for one round, in preparation for RCA detection analysis, which is performed with parallel amplification of viral subclones in HEK293A and A549. To confirm the absence of RCA at a larger scale, a subclone was amplified three times in the SF-BMAdR cell line, up to 1 L production. Briefly, the SF-BMAdR cells were cultured in PRO293s CDM or Hycell supplemented with 6 mM of glutamine. During the cell culture scale-up, cell concentration was kept between 2.5 × 10^5^ and 1.5 × 10^6^ cells/mL. On the day of infection, cells were diluted to 5 × 10^5^ cells/mL with fresh medium. The three successive infections were carried out in 6-well plates (3 mL culture), 125 mL shaker flasks (20 mL culture), and, finally, in two 2 L shaker flasks (2 × 500 mL culture). For the first two infections, cells were harvested and freeze–thawed three times three days p.i. For the last infection, the freeze/thaw cycle was performed over cell pellets resuspended in 20 mL culture. The virus was purified by CsCl two-step-gradient (1.2 and 1.4 g/cm^3^) centrifugation (59,000× *g*, 4 °C, 1 h 30) followed by continuous CsCl gradient centrifugation (59,000× *g*, 4 °C, 20 h) [24]. To quantify the viral particles (VP)/mL, the virus was diluted in a lysis solution (0.1% sodium dodecyl sulfate (SDS), 10 mM Tris-Cl pH 7.4, 1 mM EDTA) for 10 min at 56 °C. The OD260 was measured using the Nanodrop spectrophotometer (Thermo Fisher Scientific, Waltham, MA, USA) and VP/mL was calculated by multiplying the OD by the dilution factor and the extinction coefficient of 1.1 × 10^12^ [25].

The purified stock was tested for RCA presence. The A549 cells were seeded in 11 mm × 150 mm dishes at 6 × 10^6^ cells/dish. The day after, they were infected with 2 × 10^10^ VP/dish of Ad5-CMV5-NS3-5a. To observe if there were any interference effects during the RCA detection test, three dishes (control group) were also infected with 3, 5, and 10 infectious units of AdPTG3602 (a wild-type adenovirus). At 6 dpi, 5 mL fresh medium was added to each dish and the CPE was followed, up to 10 dpi. At this time, the medium and cells were harvested. After centrifugation, cells were resuspended in 3 mL of fresh medium and freeze/thawed three times. Cell lysis was centrifuged and all of the supernatant was used for infecting 11 newly seeded 150 mm dishes of A549 cells. A total of 5 mL of fresh medium was added to each dish at 6 dpi and the CPE was followed, up to 10 dpi. Cells and medium were harvested and concentrated to 1 mL and freeze/thawed three times at 10 dpi. The presence or absence of adenovirus in each sample was confirmed in the third passage by immunostaining infected A549 cells. Briefly, 96-well plates of A549 cells at a concentration of 30,000 cells/well were infected with log_10_ dilution of infected cell lysis. At 48 hpi, the cells were fixed and immunostained by a monoclonal antibody against adenovirus fiber as described previously [24].

## 3. Results

### 3.1. Cloning AdV by Limiting Dilution

Plaque purification of AdV is one of two steps in adenovirus generation that is commonly performed in an adherent cell line in the presence of FBS. Plaque purification is essential for separating wild-type adenoviruses from recombinants when adenoviruses are generated by homologue recombination in mammalian cell lines. However, if the AdV is produced by the AdEasy system (recombination happens in *E. coli* instead), plaque purification is nonmandatory, as all the produced AdVs after transfection are recombinant [26]. However, it is better to perform at least one round of plaque purification to ensure the purity and clonality of the virus and initiate vector production scale-up with a virus clone that has a confirmed GOI expression.

In this study, we developed a limiting dilution method in suspension culture without FBS to replace the plaque purification assay in order to completely remove FBS from the virus production process. The efficacy of this method for AdV clone isolation was shown by purifying an E1-deleted AdV from a stock highly contaminated with wild-type adenovirus. However, with this method, we cannot observe visible CPE in infected SF-BMAdR cells since they are in suspension. An additional amplification step in adherent cells (HEK293A) is, therefore, essential to identify the wells infected with adenoviruses. 

The number of infected wells per each ½ of the 96-well plates post immunostaining is indicated in Table 1. There is a direct correlation between the number of positive wells and the number of infectious viral particles that were used for infecting each well. At this stage, we were not able to distinguish between RCA (E1-positive) and first-generation (E1-negative) AdV, as both viruses can replicate in HEK293A cells. A comparative infection in A549 and HEK293A was needed to differentiate RCA-infected wells from E1-deleted AdV-infected wells. However, our preliminary results showed that the titer of E1-deleted AdV in infected SF-BMAdR cells with one infectious particle was low, and we could not directly use these seed stocks. Thus, an additional round of amplification in SF-BMAdR was necessary to increase the virus’ titer before comparing their CPE in A549 and HEK293A. Table 2 shows a few examples of how the result is interpreted. For example, we determined that both samples #1 and #2 contain RCA as the virus replicated in both cell lines. We determined that samples #3 and #4 are E1-deleted AdV, as they replicated only in HEK293A but not A549.

On some occasions, when the virus titer was very high (sample #4), a cytotoxic effect could be detected in the first dilution of A549. This is caused by infecting the cells with a very high multiplicity of infection (MOI). Samples like #5 and #6 are excluded from our analysis because they did not show CPE in either cell line with dilution higher than 10^−2^ and, therefore, their comparison in both cell lines was not meaningful. Of over 74 amplified adenoviruses, 39 were E1-deleted AdV and 35 were RCA with a ratio of 1.1:1, which is roughly the same as the ratio of the mixed stock for limiting dilution that we started with (1:1) (Table 1).

### 3.2. Generation of rAdV in the Absence of FBS and RCA

To have an entire process of AdV generation and production devoid of FBS and RCA, we employed an alternative cell line, the SF-BMAdR, to replace HEK293A cells at the stage of transfection. Ad5-CMV5-NS3-5a was generated in the SF-BMAdR cell line in the absence of FBS. Transfection of six-well plates with 3 μg of DNA has generated two times more AdV than transfection with 5 μg of DNA. The supernatant of infected cells in both transfections was subject to two rounds of limiting dilution. All 12 of the selected clones that underwent the first round of limiting dilution were E1-deleted AdV. One clone from each transfection was selected for the second round of limiting dilution. Again, 12 subclones were selected in total and, after one cycle of amplification in SF-BMAdR cells, followed by parallel amplification in HEK293A and A549, all subclones were characterized as E1-deleted AdV.

**Figure 2:**
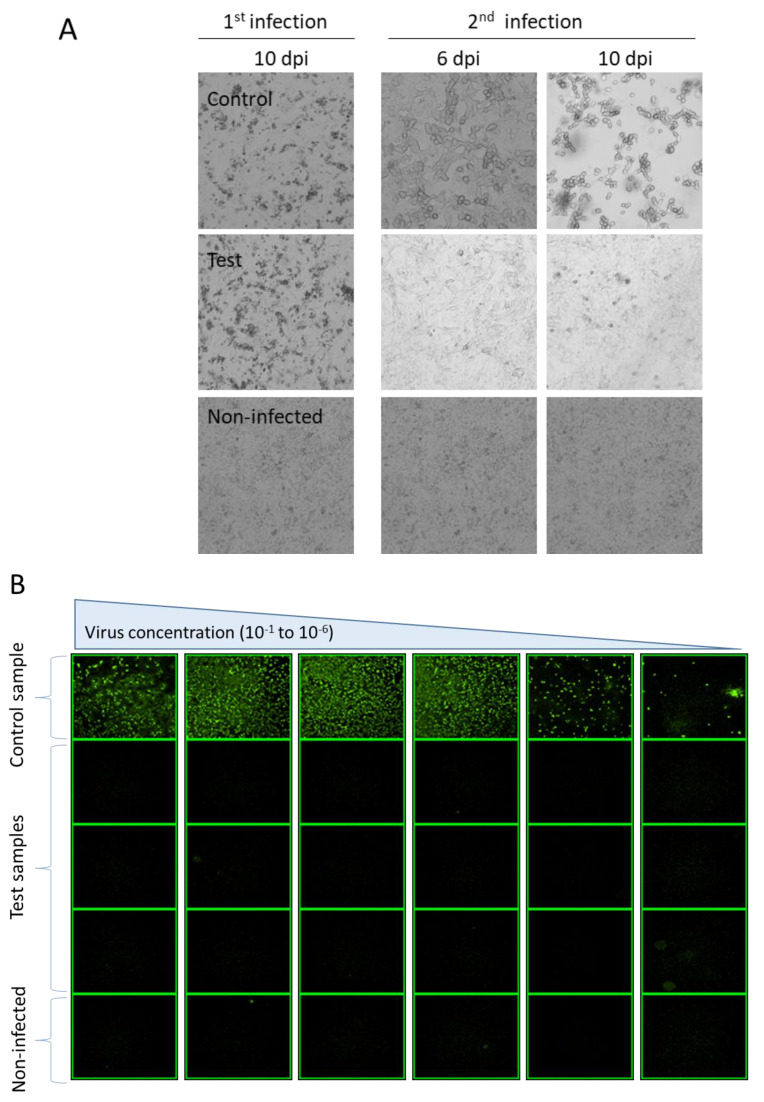
RCA detection in A549 cells. (**A**) Presence of RCA was evaluated in purified virus stock by two successive passages in A549 cells followed by the immunostaining over the third passage. In the second passage, the dishes in the control group showed CPE since 6 dpi (dish infected with 3 IU of wild-type shown) but no CPE was detected in the eight test dishes until the end of the experiment. (**B**) For immunostaining, the A549 cells were infected with different dilutions of cell lysis harvested from the second amplification. Only one photo from each virus dilution is shown. The first row shows the control group in the first passage co-infected with 3 IU of wild-type adenovirus. The second to fourth rows show three out of eight test dishes infected with only E1-deleted AdV at the first passage. The last row shows noninfected cells.

To confirm the absence of RCA, one subclone of Ad5-CMV5-NS3-5a was amplified three times in SF-BMAdR. The final 1L stock was purified. RCA detection was performed in A549 cells. At the second passage in A549 cells, the CPE was observed in all the three samples of the control group that were co-infected with 3, 5, and 10 infectious units of wild-type adenovirus but not in the eight dishes infected with only recombinant adenovirus (test group), as expected (Figure 2A). No interference was detected when cells were infected with an MOI of 1666 VP/cell of AdV (2 × 10^10^ VP/dish) and 3 to 10 ifu/dish of wild-type adenovirus. The immunostaining of infected cells confirmed that the CPE observed in the control group was caused by the replication of adenovirus. No CPE or immunostaining-positive cells were detected in the test group (infected cells with only E1-deleted AdV) (Figure 2B).

## 4. Discussion

The limiting dilution method that we developed in this paper was able to isolate E1-deleted AdV from a viral stock highly contaminated with undesirable RCA. Fortunately, in real-world applications, the level of contamination is usually much lower. This means our limiting dilution method has the potential to be even more effective.

Regardless of the method used for AdV isolation, whether it is plaque purification or limiting dilution, we could not exclude the possibility of having more than one AdV infecting the same cell or neighbouring cells (in plaque assay) or well (in limiting dilution). This could happen especially when the wells are infected with more than one infectious particle per well. However, the possibility of this happening is much lower at a higher dilution. In our limiting dilution method, if a well is infected simultaneously with an AdV and an RCA, it is identified as RCA and it will be excluded from the next step. In the case of infecting the same well with two AdVs, if the clonality of the AdV stock is imperative, another round of limiting dilution is recommended. Infecting more wells with a higher dilution in the first round of limiting dilution can be an alternative to performing a second round of limiting dilution.

The unavailability of cell lines that prevent the formation of RCA or the inability to change the AdV production process in adherent or suspension variants of the HEK293 cell line can potentially lead to RCA formation at the later stages of production. The limiting dilution method described in this paper can also be used as an alternative to plaque purification to rescue a well-characterized E1-deleted AdV stock contaminated with RCA. Specifically, the method can purify AdV stocks when the level of RCA in the stock exceeds the acceptable level for clinical trials. In addition, the lack of FBS or animal-derived components used in this process is an additional asset. To reduce the number of 96-well plates needing to be analyzed during limiting dilution, we suggest titrating the virus seed in order to infect the wells with less than 1 infectious unit per well. We used the immunostaining method for the detection of AdV-infected wells during limiting dilution to decrease the incubation period. However, immunostaining is not indispensable and, by extending the incubation period by 5–7 days, CPE will become apparent in the infected wells. 

To completely remove FBS from the entire virus generation and production process, we studied the feasibility of generating AdV by transfection of SF-BMAdR cells. The virus titer produced in SF-BMAdR cells was lower than those produced in HEK293A cells. This is due to the lower transfection efficiency of large plasmids in A549-derived cells (such as SF-BMAdR cells) compared to HEK293A cells [27]. However, this is not a significant issue since we only need tens or hundreds of infectious virus particles to start the limiting dilution. If the production of the AdV stock originates from a pool of transfected cells without prior cloning, one additional passage is needed to have a high titer seed stock for scale-up. 

The possible presence of RCA was evaluated in the purified virus stock by two successive passages in A549 cells followed by immunostaining over the third passage. In total, 9.6 × 10^7^ cells were infected with 1.6 × 10^11^ VP of AdV resulting in an MOI of 1666 VP/cell. No interference for replication of RCA was reported in the presence of up to 2000 particles per cell of E1-deleted AdV [28]. We did not detect any CPE after the first and second passage in any of the test dishes. However, all three dishes in the control group showed CPE at the second passage (but not at the first passage). Consequently, at least two passages are needed to confirm the presence of RCA. We used a conventional cell-culture-based RCA assay for detecting RCA, and the assay needs 3 weeks to complete. However, the infectivity PCR-based RCA assays [29] are faster methods that can reach the same level of sensitivity in a shorter period of time. Immunostaining was performed in our study to confirm that CPE was indeed caused by adenovirus and to further increase the sensibility of the assay, in the case that we missed a weak CPE (difficult to observe by eye) at the second passage. The test group dishes were infected in total with 1.6 × 10^11^ VP. As we were able to detect CPE caused by as low as 3 infectious units of wild-type adenovirus without interference in all the control group samples, our RCA detection method reached levels of sensitivity of 1 IU of RCA in 5.33 × 10^10^ VP, which is slightly more sensitive than the FDA recommendation of RCA in biological materials (1 IU of RCA in 3 × 10^10^ VP).

## 5. Conclusions

The method developed in this study allows us to generate clones of recombinant adenovirus in the absence of FBS and devoid of RCA. It thus improves the safety for clinical applications of AdV. Also, this limiting dilution method allows us to rescue well-characterized E1-deleted AdV stocks contaminated with RCA and decreases the amount of time required for the generation and characterization of new viral stocks.

## Figures and Tables

**Table 1:** The number of wells which were identified as either E1-deleted AdV or RCA at each concentration of the mixed seed stock. The positive wells are either scored as RCA or E1-deleted AdV or excluded from analysis. The last column presents the sum of all the analyzed wells.

Infectious Dose (ifu)/Well (SF-BMAdR)	5	2.5	1.25	0.63	0.31	0.16	Total of All Wells
Total positive wells in FFA (HEK293A)	38	27	14	7	5	1	92
Amplifed wells in SF-BMAdR cells	35	27	14	7	5	1	89
RCA (amplified in HEK293A & A549 cells)	16	10	7	2	0	0	35
AdV (amplified only in HEK293A)	13	11	6	3	5	1	39
Exclueded (Not amplified or had low titer)	6	6	1	2	0	0	15

**Table 2:** A comparative analysis of AdV (E1-negative) and RCA (E1-positive) in HEK293A and A549 cell lines. The E1-deleted AdV replicates only in HEK29A but RCA replicates in both cell lines.

	Cell Lines											
	HEK-293A	A549	HEK-293A	A549	HEK-293A	A549	HEK-293A	A549	HEK-293A	A549	HEK-293A	A549
	#1		#2		#3		#4		#5		#6	
Log10 dilution 10^−1^ to 10^−8^	+	+	+	+	+	−	+	+	+	−	−	−
	+	+	+	+	+	−	+	−	−	−	−	−
	+	+	+	+	+	−	+	−	−	−	−	−
	+	+	−	−	+	−	+	−	−	−	−	−
	+	+	−	−	+	−	+	−	−	−	−	−
	+	+	−	−	−	−	+	−	−	−	−	−
	+	−	−	−	−	−	−	−	−	−	−	−
	−	−	−	−	−	−	−	−	−	−	−	−
	**Interpretation**											
	RCA		RCA		AdV		AdV		Excluded		Excluded	

## Data Availability

The data presented in this study are available in the current article. The raw data that support the findings of this study are available on request from the corresponding author.

## References

[1] Danthinne X., Imperiale M.J. (2000). Production of First Generation Adenovirus Vectors: A Review. Gene Ther..

[2] Kratzer R.F., Kreppel F. (2017). Production, Purification, and Titration of First-Generation Adenovirus Vectors. Methods Mol. Biol..

[3] Tsilingiris D., Vallianou N.G., Karampela I., Muscogiuri G., Dalamaga M. (2022). Use of Adenovirus Type-5 Vector Vaccines in COVID-19: Potential Implications for Metabolic Health?. Minerva Endocrinol..

[4] World Health Organization COVID-19 Vaccine Tracker and Landscape. https://www.who.int/publications/m/item/draft-landscape-of-covid-19-candidate-vaccines.

[5] Graham F.L., Smiley J., Russell W.C., Nairn R. (1977). Characteristics of a Human Cell Line Transformed by DNA from Human Adenovirus Type 5. J. Gen. Virol..

[6] Louis N., Evelegh C., Graham F.L. (1997). Cloning and Sequencing of the Cellular-Viral Junctions from the Human Adenovirus Type 5 Transformed 293 Cell Line. Virology.

[7] Lochmüller H., Jani A., Huard J., Prescott S., Simoneau M., Massie B., Karpati G., Acsadi G. (1994). Emergence of Early Region 1-Containing Replication-Competent Adenovirus in Stocks of Replication-Defective Adenovirus Recombinants (Delta E1 + Delta E3) during Multiple Passages in 293 Cells. Hum. Gene Ther..

[8] Hehir K.M., Armentano D., Cardoza L.M., Choquette T.L., Berthelette P.B., White G.A., Couture L.A., Everton M.B., Keegan J., Martin J.M. (1996). Molecular Characterization of Replication-Competent Variants of Adenovirus Vectors and Genome Modifications to Prevent Their Occurrence. J. Virol..

[9] Zhu J., Grace M., Casale J., Chang A.T., Musco M.L., Bordens R., Greenberg R., Schaefer E., Indelicato S.R. (1999). Characterization of Replication-Competent Adenovirus Isolates from Large-Scale Production of a Recombinant Adenoviral Vector. Hum. Gene Ther..

[10] Food and Drug Administration (2020). Chemistry, Manufacturing, and Control (CMC) Information for Human Gene Therapy Investigational New Drug Applications (INDs) Guidance for Industry.

[11] Farson D., Tao L., Ko D., Li Q., Brignetti D., Segawa K., Mittelstaedt D., Harding T., Yu D.-C., Li Y. (2006). Development of Novel E1-Complementary Cells for Adenoviral Production Free of Replication-Competent Adenovirus. Mol. Ther..

[12] Gilbert R., Guilbault C., Gagnon D., Bernier A., Bourget L., Elahi S.M., Kamen A., Massie B. (2014). Establishment and Validation of New Complementing Cells for Production of E1-Deleted Adenovirus Vectors in Serum-Free Suspension Culture. J. Virol. Methods.

[13] Kim J.S., Lee S.H., Cho Y.S., Park K., Kim Y.H., Lee J.H. (2001). Development of a Packaging Cell Line for Propagation of Replication-Deficient Adenovirus Vector. Exp. Mol. Med..

[14] Kovesdi I., Hedley S.J. (2010). Adenoviral Producer Cells. Viruses.

[15] Howe J.A., Pelka P., Antelman D., Wilson C., Cornell D., Hancock W., Ramachandra M., Avanzini J., Horn M., Wills K. (2006). Matching Complementing Functions of Transformed Cells with Stable Expression of Selected Viral Genes for Production of E1-Deleted Adenovirus Vectors. Virology.

[16] Xu Q., Arevalo M.T., Pichichero M.E., Zeng M. (2006). A New Complementing Cell Line for Replication-Incompetent E1-Deleted Adenovirus Propagation. Cytotechnology.

[17] Fallaux F.J., Bout A., van der Velde I., van den Wollenberg D.J., Hehir K.M., Keegan J., Auger C., Cramer S.J., van Ormondt H., van der Eb A.J. (1998). New Helper Cells and Matched Early Region 1-Deleted Adenovirus Vectors Prevent Generation of Replication-Competent Adenoviruses. Hum. Gene Ther..

[18] Gao G.P., Engdahl R.K., Wilson J.M. (2000). A Cell Line for High-Yield Production of E1-Deleted Adenovirus Vectors without the Emergence of Replication-Competent Virus. Hum. Gene Ther..

[19] Xie L., Pilbrough W., Metallo C., Zhong T., Pikus L., Leung J., Auniņs J.G., Zhou W. (2002). Serum-Free Suspension Cultivation of PER.C6(R) Cells and Recombinant Adenovirus Production under Different PH Conditions. Biotechnol. Bioeng..

[20] Gstraunthaler G., Lindl T., van der Valk J. (2013). A Plea to Reduce or Replace Fetal Bovine Serum in Cell Culture Media. Cytotechnology.

[21] Young K.G., Haq K., MacLean S., Dudani R., Elahi S.M., Gilbert R., Weeratna R.D., Krishnan L. (2018). Development of a Recombinant Murine Tumour Model Using Hepatoma Cells Expressing Hepatitis C Virus Nonstructural Antigens. J. Viral. Hepat..

[22] Haq K., Jia Y., Elahi S.M., MacLean S., Akache B., Gurnani K., Chattopadhyay A., Nazemi-Moghaddam N., Gilbert R., McCluskie M.J. (2019). Evaluation of Recombinant Adenovirus Vectors and Adjuvanted Protein as a Heterologous Prime-Boost Strategy Using HER2 as a Model Antigen. Vaccine.

[23] Oualikene W., Lamoureux L., Weber J.M., Massie B. (2000). Protease-Deleted Adenovirus Vectors and Complementing Cell Lines: Potential Applications of Single-Round Replication Mutants for Vaccination and Gene Therapy. Hum. Gene Ther..

[24] Elahi S.M., Nazemi-Moghaddam N., Gadoury C., Lippens J., Radinovic S., Venne M.-H., Marcil A., Gilbert R. (2021). A Rapid Focus-Forming Assay for Quantification of Infectious Adenoviral Vectors. J. Virol. Methods.

[25] Mittereder N., March K.L., Trapnell B.C. (1996). Evaluation of the Concentration and Bioactivity of Adenovirus Vectors for Gene Therapy. J. Virol..

[26] Reddy P.S., Ganesh S., Hawkins L., Idamakanti N. (2007). Generation of Recombinant Adenovirus Using the Escherichia Coli BJ5183 Recombination System. Methods Mol. Med..

[27] Søndergaard J.N., Geng K., Sommerauer C., Atanasoai I., Yin X., Kutter C. (2020). Successful Delivery of Large-Size CRISPR/Cas9 Vectors in Hard-to-Transfect Human Cells Using Small Plasmids. Commun. Biol..

[28] Murakami P., Havenga M., Fawaz F., Vogels R., Marzio G., Pungor E., Files J., Do L., Goudsmit J., McCaman M. (2004). Common Structure of Rare Replication-Deficient E1-Positive Particles in Adenoviral Vector Batches. J. Virol..

[29] Schalk J.A.C., de Vries C.G.J.C.A., Orzechowski T.J.H., Rots M.G. (2007). A Rapid and Sensitive Assay for Detection of Replication-Competent Adenoviruses by a Combination of Microcarrier Cell Culture and Quantitative PCR. J. Virol. Methods.

